# Current practice of stress ulcer prophylaxis in a surgical patient cohort in a German university hospital

**DOI:** 10.1007/s00423-021-02325-3

**Published:** 2021-09-14

**Authors:** Julia Rauch, Maciej Patrzyk, Claus-Dieter Heidecke, Tobias Schulze

**Affiliations:** 1 Department of General Surgery, Visceral, Thoracic and Vascular Surgery, Universitätsmedizin Greifswald, Ferdinand-Sauerbruch-Straße, 17475 Greifswald, Germany; 2 Present Address: IQTIG – Institut für Qualitätssicherung und Transparenz im Gesundheitswesen, Berlin, Germany

**Keywords:** Stress ulcer, Antacids, Perioperative care, Stress ulcer prophylaxis

## Abstract

**Introduction:**

Stress ulcer prophylaxis (SUP) has been a widespread practice both in intensive care units (ICU) and internal wards at the beginning of the twenty-first century. Clinical data suggests an important overuse of acid suppressive therapy (AST) for this indication. Data on current clinical practice of SUP in surgical patients in a non-ICU setting are spares. In the light of a growing number of reports on serious side effects of AST, this study evaluates the use of AST for SUP in a normal surgical ward in a German university hospital.

**Methods:**

Between January 2016 and June 2016, SUP was analysed retrospectively in 1132 consecutive patients of the Department of Surgery of the Universitätsmedizin Greifswald.

**Results:**

The patients managed with and without SUP were similar with respect to demographic data and treatment with anticoagulants, SSRI and glucocorticoids. Patients with SUP were treated more frequently by cyclooxygenase inhibiting drugs (NSAID, COX2-inhibitors), were more frequently treated in the intermediated care unit and had a longer hospital stay. Risk factors for the development of stress ulcers were similarly present in patient groups managed with and without SUP. About 85.7–99.6% of patients were given SUP without an adequate risk for stress ulcer development, depending on the method used for risk assessment.

**Discussion:**

Still today, SUP is widely overused in non-ICU surgical patients. Information campaigns on risk factors for stress ulcer development and standard operating procedures for SUP are required to limit potential side effects and increased treatment costs.

**Supplementary Information:**

The online version contains supplementary material available at 10.1007/s00423-021-02325-3.

## Introduction

The occurrence of upper intestinal bleeding in seriously ill surgical patients has been initially reported more than 150 years ago [1]. The incidence of stress ulcer disease varied over the time and with the patient population considered. With the broad introduction of fibrotic endoscopy in clinical medicine in the 1970s, the presence of gastric mucosal lesions was detected in up to 100% of severely injured patients, resulting in clinically significant bleeding in 22% of this population [2]. This is in accordance with the incidence of overt gastrointestinal (GI) bleeding in up to 25% of intensive care unit patients reported in 1978 by Hastings et al. [3]. Mortality from gastroduodenal ulceration in this setting was reported to be as high as 58% in this decade [4]. The high incidence and the potentially live threatening consequences of this condition resulted in the development of various pharmacological measures aiming at the prevention of stress ulcer disease. While antacids were the mainstay of pharmacologic treatment in the mid-twentieth century, a highly effective medical treatment became available with introduction of H2-receptor antagonists (H2RA) in 1978 and of the proton pump inhibitors (PPI) in 1988 [5]. In the light of the potentially severe sequel of stress-induced ulcer disease, use of pharmacologic stress ulcer prophylaxis was a routine treatment in the majority of intensive care units at the beginning of the twenty-first century [6]. At this time, clinically important GI bleeding occurred in 3.5% of ICU patients, resulting in a 20–30% increase of mortality [7]. During the following years, diagnostic and therapeutic options and the process of care in intensive medicine further improved [8, 9] resulting in decreased incidence of GI bleeding in this patient population. In 2015, an international 7-day inception cohort study found an episode of clinically important GI bleeding in 2.7% of ICU patients, and the 90-day mortality was not increased in the confounder-adjusted analysis [10].

In contrast, stress ulcer formation with consecutive GI bleeding outside of the ICU setting appears to be a rare event, occurring in 0.26–0.27% of non-ICU medical patients [11]. Notwithstanding this fact, the current practice of pharmacological SUP in the ICU population has been largely extrapolated to non-critical ill patients in non-ICU wards and in skilled nursing facilities. Thus, up to 88.5% of patients in internal medicine wards and 65.3% of long-term care residents on AST for SUP receive these medications without reproducible indication [12, 13]. While many investigators confirm this observation in internal medicine and general medicine patients [12, 14–18], data on the incidence of inappropriate use of AST for SUP in surgical patients is sparse. A literature search revealed only two publications reporting on this issue. Bez et al. found that of all patients receiving AST for SUP in a general surgery ward, 79% presented no risk factors for stress ulcers [19]. Parente et al. described an inappropriate use of AST for SUP in 67% of surgical patients, an incidence comparable to an internal medicine cohort considered in the same study [20]. Growing concerns on potentially severe side effect of AST [21, 22] challenge the reported practice of SUP in non-ICU surgical patients. The present work addresses the question whether more than 30 years of ongoing controversy on the adequacy of SUP in this patient population [23] has elicited a modification of clinical practice in the Department of General Surgery, Visceral, Thoracic and Vascular Surgery of the University Medical Centre in the north of Germany.

## Materials and methods

### Setting, data collection and patients

**Fig. 1:**
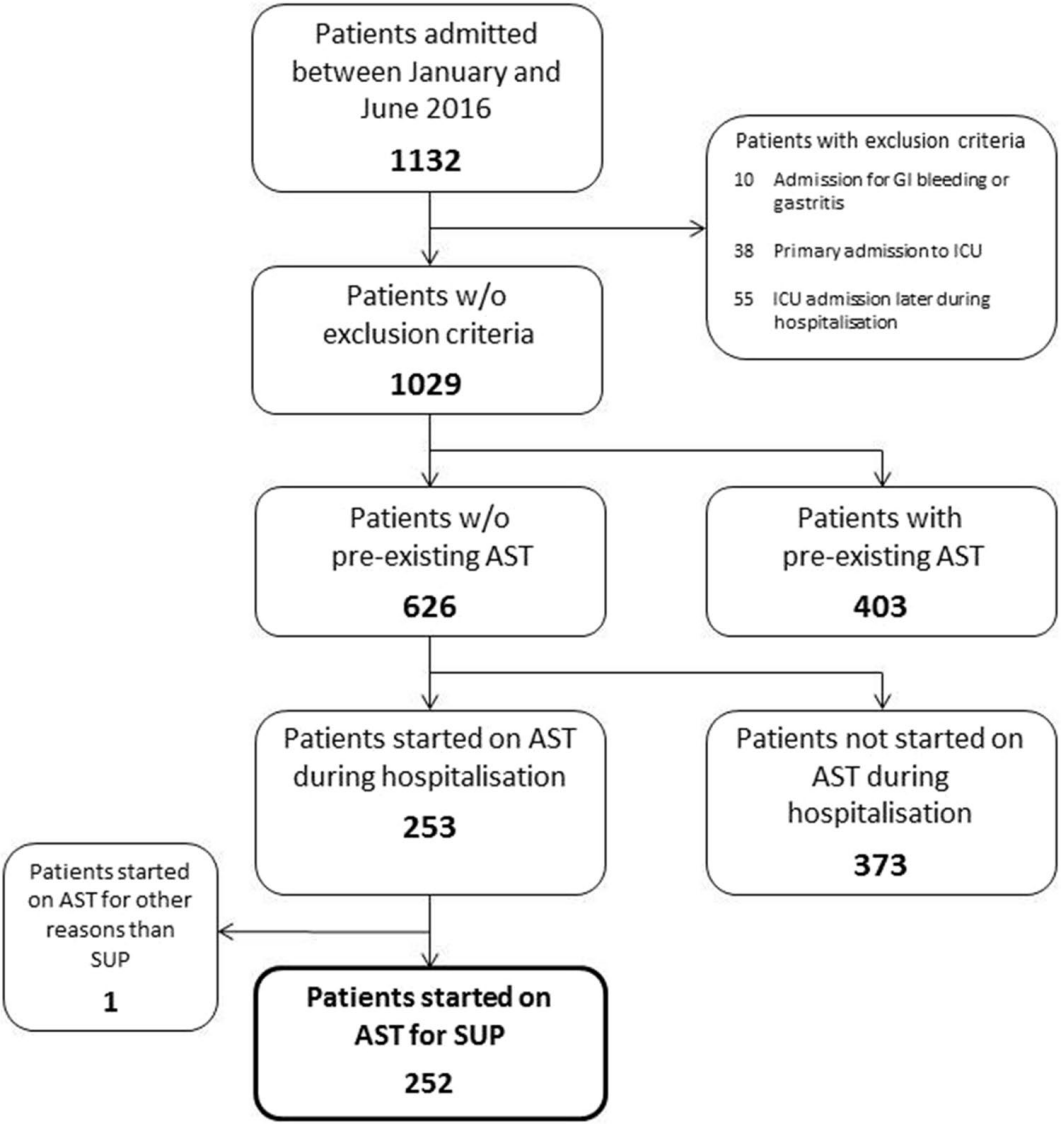
Study population

In this retrospective observational study, data was collected from 1132 consecutive patients aged > 18 years admitted to the Department of General Surgery, Visceral, Thoracic and Vascular Surgery between January 2016 and June 2016 was performed. The study design was approved by the clinical ethics committee of the Universitätsmedizin Greifswald. Patients admitted to the hospital for peptic ulcer disease and gastritis, those who were primarily admitted to the ICU and those who were admitted to the ICU during the stay in the hospital were excluded from the analysis. Patient admitted twice or more frequently were not re-included. Patients with other documented indications for perioperative de novo AST administration were as well excluded from the study (Fig. 1).

Data was collected from the paper-based patient records using a computer-based access form and included demographic data; data concerning previous illnesses including personal history of gastrointestinal bleeding, ulcer disease, GERD and dyspepsia; data concerning the motif for the present admission; treatment-associated data; information on gastrointestinal bleeding episodes during the hospital stay and medication during hospitalisation and prescribed in the hospital discharge letter. Detailed information on AST started during the index admission were retrieved, including the type of medication, the setting of prescription (intermediate care/normal care ward), the moment of prescription (preoperative/postoperative) and the duration of AST administration. Patients discharged with AST were contacted by letter in order to evaluate the duration AST prescribed in the discharge letter. Data for the evaluation of the individual bleeding risk were chosen according to the literature [11, 19, 24, 25].

### Risk evaluation

In order to evaluate the individual risk for gastrointestinal bleeding, six risk constellations for gastrointestinal bleeding were defined according to the current literature [24, 25]: risk constellation 1, antiplatelet agent combined with one of the factors such as age > 60 years, systemic glucocorticoids, personal history of ulcer disease or severe systemic disease (ASA ≥ 3); risk constellation 2, anticoagulant medication combined with ≥ 2 of the risk factors listed above; risk constellation 3, combination of at least two anticoagulant or antiplatelet agents; risk constellation 4, long-term therapy with NSAID; risk constellation 5, combination of serotonin reuptake inhibitors with NSAID or COX inhibitors; and risk constellation 6, combination of COX inhibitors with anticoagulants or antiplatelet agents. Pharmacological stress ulcer prophylaxis was considered indicated when at least one risk factor as described above was present. Alternatively, risk factors were defined as done by Bez et al. [19]. Presence of one risk factor according to Bez was considered as justifying pharmacological SUP. Finally, the clinical risk scoring system for nosocomial bleeding (CRSSNB) developed by Herzig et al. was used to determine the risk for a bleeding episode as described by the authors [11]. Risk groups for gastrointestinal bleeding were defined as previously reported [11]. A CRSSNB ≥ 10 was considered as indication for SUP [11].

### Statistical analysis

Categorical variables are described using frequency. Continuous variables are described using the non-parametric measures median and interquartile range. For the comparison of categorical variables, chi-square tests were applied; for small data, the Fischer’s exact test was used. For the continuous variables, the t-test was used after verification of Gaussian distribution by the Kolmogorov–Smirnov test. If the variables showed no Gaussian distribution, the Mann–Whitney test was applied. Statistical analysis was conducted using SPSS software version 25.0 (IBM Inc., Armonk, NY).

## Results

### Study population

Of the 1132 patients admitted to the Department of General, Visceral, Thoracic and Vascular Surgery of the University Medicine Greifswald during the study period, 103 patients presented exclusion criteria including admission for gastritis or GI bleeding, primary admission to the ICU or ICU admission later during the index hospitalisation (Fig. 1). Among the remaining 1029 patients without exclusion criteria, 403 patients had AST listed in their home medication. Of the 626 patients admitted to the hospital without pre-existing AST, 252 patients were started on SUP; one further patient was given AST for other indications during the hospital stay. Three hundred seventy-three patients received no SUP during their stay in the hospital.

### Demographic and treatment characteristics of patients with and without SUP during hospitalisation

Table 1 shows demographic data and home medication of the patients admitted to the hospital without pre-existing AST. The patient group started on SUP after admission was compared with those patients managed without SUP. Both patient groups were similar with respect to age, sex and body mass index. Patients with SUP had a tendency towards higher ASA levels; however, this difference was not statistically significant. NSAIDs and COX2-inhibitors (COX2-I) were taken more frequently by patients being started on SUP; however, their absolute number was very low in both groups (with and without SUP). Other home medication (glucocorticoids, SSRI) predisposing patients to the development of gastric ulcers were pre-existing in both groups to a similar extent. Medication interfering with blood coagulation was similarly present in patients put on SUP or not.

**Table 1:** Demographic data of patients admitted to the hospital without previous acid suppressive medication. Patients being started on SUP during hospitalisation are compared to those without SUP. *ASA* ASA Physical Status Classification System, *BMI* body mass index, COX1-I = COX2-inhibitors

	Patients admitted to the hospital without ASM **with SUP** during hospitalisation (*n* = 252)	Patients admitted to the hospital without ASM **without SUP** during hospitalisation (*n* = 373)	*p*
Age			
Mean ± SD	56.6 ± 16.5	53.8 ± 17.2	0.43
Sex			
Male (%)	139 (55.2)	226 (60.6)	0.19
Female (%)	113 (44.8)	147 (39.4)	
BMI ± SD	27.6 ± 5.7	28.0 ± 6.1	0.71
ASA			
I	39 (15.5)	66 (17.7)	
II	132 (52.4)	218 (58.4)	0.05
III	75 (29.8)	87 (23.3)	
IV	6 (2.4)	2 (0.5)	
Medication at admission to hospital (%)			
NSAID/COX2-I			
NSAID	3 (1.2)	1 (0.3)	
COX2-I	6 (2.4)	1 (0.3)	0.02
None	243 (96.4)	371 (99.4)	
Glucocorticoids			
Yes	6 (2.4)	3 (0.8)	0.17
No	246 (97.6)	370 (99.2)	
SSRI			
Yes	13 (5.2)	9 (2.4)	0.08
No	239 (94.8)	364 (97.6)	
Anticoagulation when admitted to hospital (%)			
None	192 (76.2)	284 (76.1)	
Plasmatic	10 (4.0)	17 (4.6)	0.95
Antiplatelet	47 (18.7)	69 (18.5)	
Both	3 (1.2)	3 (0.8)	

Treatment characteristics of patients admitted to the hospital without pre-existing AST are summarised in Table 2. Patients started on SUP stayed in the hospital for 8.3 ± 10.5 days, while patients without SUP were discharged after 3.0 ± 3.8 days (*p* < 0.05). Patients sojourning in the intermediate care unit (IMC) during their hospitalisation were more likely to be started on SUP than patients who did not (65.5% versus 8.3%, *p* < 0.05). Interestingly, patients in the thoracic surgery branch of the department were more likely to be put on SUP (72.0%) than those in the vascular surgery (41.9%), the visceral surgery (38.8%) or general surgery branch (22.0%). There was no difference between patients managed by conservative, interventional or surgical treatment concepts with respect to SUP initiation.

**Table 2:** Treatment data of patients admitted to the hospital without previous acid suppressive medication. Patients being started on SUP during hospitalisation are compared to those without SUP

	Patients admitted to the hospital without ASM **with SUP** during hospitalisation (*n* = 252)	Patients admitted to the hospital without ASM **without SUP** during hospitalisation (*n* = 373)	*p*
Length of hospital stay (days)			
Mean ± SD	8.3 ± 10.5	3.0 ± 3.8	< 0.05
Stay in the intermediate care unit (%)			
Yes	165 (65.5)	31 (8.3)	< 0.05
No	87 (34.5)	342 (91.7)	
Specialty (%)			
General surgery	35 (13.9)	128 (34.3)	
Visceral surgery	118 (46.8)	168 (45.0)	< 0.05
Thoracic surgery	59 (23.4)	23 (6.2)	
Vascular surgery	39 (15.5)	54 (14.5)	
Therapy (%)			
Conservative	27 (10.9)	60 (16.2)	
Interventional	23 (9.3)	41 (11.1)	0.11
Surgical	198 (79.8)	270 (72.8)	

Table 3 shows the admission diagnosis of patients without AST in the home medication. Patients with thoracic pathologies were more likely to be set on SUP than patients suffering vascular or visceral pathologies. Within the group “visceral pathologies/pathologies affecting the abdominal wall”, patients with malignant diseases of the colorectum and hepatobiliary tract were more likely to receive SUP than patients with benign diseases. In the same category, patients with intraabdominal pathologies were more likely to receive SUP than those with diseases affecting the abdominal wall. Finally, all patients without previous AST and suffering from diseases of the stomach received SUP. Within the group with vascular pathology group, patients with diseases affecting the venous system were less likely to be put on SUP (Supplementary Table 1).

**Table 3:** Diagnosis at discharge of patients admitted to the hospital without previous acid suppressive medication. Patients being started on SUP during hospitalisation are compared to those without SUP. Diagnoses were classified into “thoracic pathologies”, “vascular pathologies”, “visceral pathologies and pathologies concerning the abdominal wall” as well as “others”. Frequencies were compared between patients with and without SUP. Detailed diagnostic information within the diagnostic groups is shown in supplementary table 1

	Patients admitted to the hospital without ASM **with SUP** during hospitalisation (*n* = 252)	Patients admitted to the hospital without ASM **without SUP** during hospitalisation (*n* = 373)	*p*
Thoracic pathologies	59 (23.4)	23 (6.2)	
Vascular pathologies	39 (15.5)	54 (14.5)	
Visceral pathologies/pathologies of the abdominal wall	149 (59.1)	295 (79.1)	0.0
Others	5 (2.0)	1 (0.3)	

### Risk for the development of stress ulcer disease and upper GI bleeding in patients without pre-existing AST

Based on the presence of individual risk factors in combination with concurrent risk medication, six risk constellations for gastrointestinal bleeding were defined according to the current literature [24, 25]. Interestingly, none of these risk constellations were present in the majority of patients both started or not on SUP (85.7% versus 90.3%) (Table 4). Only 35 patients among those with SUP and 36 patients among those without SUP had one risk constellation (with SUP: 13.9% versus without SUP: 9.7%). One patient presented more than one risk constellation in the patient group with SUP (with SUP: 0.4% versus without SUP: 0.0%). These differences were not statistically significant (*p* = 0.12).

**Table 4:** Presence or absence of risk factors for gastrointestinal bleeding in patients admitted to the hospital without previous acid suppressive medication. Patients being started on SUP during hospitalisation are compared to those without SUP

	Patients admitted to the hospital without ASM **with SUP** during hospitalisation (*n* = 252)	Patients admitted to the hospital without ASM **without SUP** during hospitalisation (*n* = 373)	*p*
Number of pharmacological risk factors (%)			
0	216 (85.7)	337 (90.3)	
1	35 (13.9)	36 (9.7)	
2	1 (0.4)	0	
3	0	0	0.12
4	0	0	
5	0	0	
6	0	0	
Presence of risk factors according to Bez et al			
0	238 (94.4)	360 (96.5)	
1	13 (5.2)	13 (3.5)	0.28
> 1	1 (0.4)	0	
CRSSNB according to Herzig et al			
< 6	153 (60.7)	270 (72.4)	
≥ 6	76 (30.2)	87 (23.3)	
≥ 8	22 (8.7)	16 (4.3)	< 0.05
≥ 10	1 (0.4)	0	
≥ 12	0	0	

In 1998, several risk factors for nosocomial gastrointestinal bleeding were identified and published by the American Society of Health-System Pharmacist in patients hospitalised in the ICU [19, 26]. As expected in the non-ICU setting considered in this study, none of these risk factors were present in the majority of patients both in the group with and without SUP (94.4% versus 96.5%, respectively). One risk factor was present in 13 patients in each group (with SUP: 5.2% and without SUP: 3.5%). Only one patient had more than 1 risk factor, and this patient received SUP. The differences between the groups were not statistically significant.

Herzig et al. developed a scoring system for gastrointestinal bleeding in non-critically ill patients. Depending on the presence of individual risk factors, a maximum score of 12 points for the highest risk represents the highest risk for nosocomial bleeding. The authors consider a risk score of at least 10 to be an acceptable indication for the initiation of SUP [11]. Only one patient in the group with SUP reached a risk score of 10 (0.4%). However, patients put on SUP had significantly higher risk scores compared to patients without SUP (Table 4).

### Gastrointestinal bleeding and esophagogastroduodenoscopy during hospitalisation

Of those patients without pre-existing AST, only 1 patient developed overt gastrointestinal bleeding (0.4%). This patient belonged to the group receiving SUP (Table 5). Upper gastrointestinal tract endoscopy was performed in 9 patients in the group receiving SUP and in 2 patients without SUP. However, suspicion of gastrointestinal bleeding was the indication for only one esophagogastroduodenoscopy in the SUP group (Table 5).

**Table 5:** Gastrointestinal bleeding and upper GI tract endoscopy in patients admitted to the hospital without previous acid suppressive medication. Patients being started on SUP during hospitalisation are compared to those without SUP

	Patients admitted to the hospital without ASM **with SUP** during hospitalisation (*n* = 252)	Patients admitted to the hospital without ASM **without SUP** during hospitalisation (*n* = 373)	*p*
Gastrointestinal bleeding			
Stress-ulcer related	1 (0.4)	0	
Other reasons	0	0	0.403
None	251 (99.6)	373 (100)	
Esophagogastroduodenoscopy			
For GI bleeding	1 (0.4)	0	
For other reasons	8 (3.2)	2	1.0
None	243 (96.4)	371 (99.5)	

### Appropriateness of SUP initiated during hospitalisation

Among the 252 patients started de novo on AST for SUP during the hospitalisation, the appropriateness of the indication was determined retrospectively. When the decision on the appropriateness of SUP was based on the presence of at least one risk constellation defined as described in materials and methods, initiation of SUP was indicated in 36 patients (14.3%). In 216 patients (85.7%), no indication for SUP could be identified based on the above mentioned criteria.

When the criteria for an increased risk of gastrointestinal bleeding under ICU conditions [26] were applied, 14 patients (5.6%) presented a least 1 risk factor. Conversely, no indication for SUP could be found in 238 patients (94.4%) based in these criteria.

Finally, when the criteria defined by Herzig et al. were applied, SUP was indicated in only 1 patient in our cohort, corresponding to 0.4%. According to these criteria, no indication could be identified in 99.6% of patients.

### Characteristics of SUP treatment during hospitalisation

In patients without any reproducible indication for SUP according to the risk constellations defined in the Materials and Methods section, this treatment was started 2.0 ± 4.2 days after admission to the hospital. In contrast, in patients presenting an indication, SUP was initiated 4.2 ± 7.4 days after admission. Accordingly, in patients receiving SUP with reproducible indication, the first dose of SUP was most frequently administered post-operatively/post-interventionally. In patients without an indication, SUP was started more often pre-operatively/pre-interventionally than in patients with indication for SUP. In patients requiring transfer to the intermediate care station (165 patients), SUP with reproducible indication was administered significantly more often than in patients without transition to the intermediate care station (29 patients (17.6%) versus 7 patients (9.1%), respectively, *p* = 0.04). In patients admitted to the intermediate care station during the hospitalisation, treatment was discontinued when patients were transferred to the normal ward in 12 patients (8.8%) of 136 cases where SUP was given without reproducible indication.

The pharmacological group most frequently used for SUP were proton pump inhibitors. There was no difference between the patients receiving SUP with and without reproducible indication (Table 6).

**Table 6:** Pharmacological groups used for SUP. Patient groups receiving SUP with and without reproducible indication were compared. *PPI* proton pump inhibitors, *H2-antagonist* histamine receptor 2 antagonist

	Patients receiving SUP **with** reproducible indication (*n* = 36)	Patients receiving SUP **without** reproducible indication (*n* = 216)	*p*
Pharmacological group used for SUP (%)			
PPI	33 (91.7)	209 (96.8)	
H2-antagonist	3 (8.3)	7 (3.2)	0.16
Both sequentially	0	0	
Others	0	0	

### Discontinuation of SUP after hospital discharge

In the majority of patients receiving SUP without reproducible indication, the treatment was discontinued on discharge from hospital (147 patients, 68.1%). In contrast, in patients with reproducible risk factors during the hospital stay, treatment was discontinued in only 58.3% (21 patients). However, 12 of the remaining patients of the latter group had risk factors justifying a continued medication with AST. Patients without risk factors and discharged from the hospital with continued AST were interviewed by questionnaire in order to assess whether and when AST was discontinued by the family practitioner (Table 7). From 70 patients contacted, 27 patients returned completed questioners. Only three of them (11.1%) still continued taking AST after more than 9 months. In the majority of cases, AST was discontinued by the family practitioner 1 to 3 months after discharge from hospital.

**Table 7:** Time to discontinuation of SUP after discharge from hospital

	Patients receiving SUP **with** reproducible indication (*n* = 3)	Patients receiving **SUP without** reproducible indication (*n* = 27)	*p*
Time elapsed until cessation of SUP (%)			
Immediately discontinued	2 (66.6)	7 (25.9)	
< 1 month	0	0	
1–3 months	0	10 (37.0)	
4–6 months	1 (33.3)	4 (14.8)	0.5
7–9 months	0	2 (7.4)	
No cessation	0	3 (11.1)	
Taken on demand	0	1 (3.7)	

### Appropriateness of AST in the pre-existing home medication

Among the 1029 patients without exclusion criteria, 403 patients (39.1%) received AST. Of those, 188 patients (46.7%) presented a reproducible indication, while no risk factors or pathologies requiring AST could be identified in 215 patients (53.3%). In both groups, AST was maintained in 100% of patients during the hospitalisation. Interestingly, in those patients without a reproducible indication for AST on admission, this medication was discontinued in 6.5% of patients on discharge, while it was continued in 93.0%.

## Discussion

Twenty years after the publication of the first report on the over-prescription of AST for the prevention of stress ulcers in non-ICU patients [27], SUP is still a widely used practice in patients on non-ICU surgical wards. Among the patients admitted without pre-existing ASM, 40.3% received SUP de novo. This is slightly less than the 54% frequency of newly started ASM in surgical patients found by Bez et al. in 2013 but comparable to that found in a mixed surgical and medical patient cohort described by Parente et al. in 2003 in surgical patients [19, 20]. In general medical ward patients, ASM for SUP is started de novo on hospital admission in 22.1–84% [15, 16, 18, 28, 29]. Thus, the frequency of SUP administration in surgical and internal medicine non-ICU wards seems to occur to a similar extent.

It is important to address the issue of the criteria on which the decision is based to prescribe SUP during hospitalisation. The comparison of patients started on SUP with those who were not showed no significant differences between demographic data of both groups. There was a tendency to higher ASA groups in patients receiving SUP. Pre-existing medication with NSAID/Cox-2 inhibitors was significantly more frequent in patients being started on SUP. However, the overall frequency of this medication was very low. Patients with de novo SUP had higher total days in hospital and required ICU monitoring more frequently than patients without SUP. These findings suggest that treating surgeons provided SUP preferentially to patients with more severe clinical courses. Accordingly, patients started on SUP had higher clinical risk scores for nosocomial GI bleeding than those not started on SUP. However, the absolute risk for gastrointestinal bleeding, as judged by the clinical risk score developed by Herzig, was very low in all but one patient in this study, ranging from 0.1 to 0.68%, not justifying SUP [11]. Our findings indicate that in clinical routine, the relative risk for the development of nosocomial gastrointestinal bleeding in patients on a normal surgical ward is correctly judged by the treating surgeon, while the absolute risk in comparison to the potential side effects of AST is largely overestimated. The usage of clinical risk scores to assess the risk for nosocomial gastrointestinal bleeding could be an appropriate tool to remediate this misjudgement. The low incidence of stress ulcer-associated gastrointestinal bleeding found in our patient cohort suggest that the low risk of stress-induced gastrointestinal bleeding found in a mixed patient cohort on medical and non-medical normal wards also applies to non-critically ill surgical patients [11].

Appropriateness of newly started ASM for SUP during hospital admission has been defined very heterogeneously in publications during the last 20 years [12, 14–20, 28]. In consequence, the comparison of the incidences of inappropriate SUP between various reports is challenging. In surgical patients, SUP without adequate indication is reported in 67.0–72.6% of patients [19, 20]. In medical patients, SUP was given without indication to 36.9–100.0%. [12, 14–18, 28, 30]. According to the only official guideline on stress ulcer prophylaxis published to date, SUP is not recommended in patients outside the ICU [26]. Thus, in a strict interpretation of this recommendation, every stress ulcer prophylaxis initiated in non-ICU wards would be inadequate. However, the authors also state that “the presence of patient risk factors for clinically important bleeding, not just admission to an intensive care unit should determine the need for a stress ulcer prophylaxis”. Risk factors can be derived from current recommendations on gastric protection in presence of risk medication [24, 25]. Moreover, risk scores published and validated in the literature can be used to assess the patient risk to develop clinically significant GI bleeding [11, 19]. Depending on the criteria used to define appropriateness, SUP initiated in the surgical collective in this study was inappropriate in 85.7–99.6% of patients. Based on the risk score of Herzig et al., the majority of our patients started on SUP (99.6%) had a risk for clinically significant bleeding of less than 0.7%. In the majority of our patients (risk score ≤ 8), the number of needed-to-treat to prevent a bleeding episode is 500 or higher. This exceeds by far the number needed to harm for nosocomial pneumonia [31]. The application of clinical risk scores may improve the risk assessment for GI bleeding in clinical routine and thus increase the appropriateness of AST use for SUP.

In the current medical literature, there is only little evidence that a specific surgical procedure requires prophylactic SUP in the non-ICU setting. In most cases, the indication for prophylactic SUP results from the risk profile of the patient based on specific medications or co-morbidities. In contrast, in bariatric surgery, prophylactic administration of AST as SUP is a widely approved part of perioperative treatment protocols due to the high frequency of marginal ulcers occurring after this kind of surgery [32–34]. However, prophylactic perioperative use of AST has to be distinguished from the therapeutic perioperative administration of AST for the treatment of postoperative functional dyspepsia or manifest gastritis or pre-existing pathologies [35].

In the majority of recent reports as well as in our study, PPI were the most frequently used drug class for SUP [12, 15, 17, 18, 28]. Although PPI have been considered to be a very safe drug after their introduction to the market, there is increasing controversy concerning potentially severe side effects of these molecules, including bacterial gastroenteritis [22], acute interstitial nephritis [36], vitamin B deficiency [37], community and hospital acquired pneumonia, dementia, osteoporosis and electrolyte disturbances (for review see [21]). There have also been epidemiological reports on long-term PPI use and increased risk of certain cancers, e.g. gastric cancer and pancreatic cancer [38–40]. Even if there is still considerable controversy whether or not the risk increase for certain side effects is sufficiently important to be clinically relevant, a valid indication should be verified before starting PPI administration. This was not the case in the vast majority of patients that have been started on SUP de novo during their hospital admission. Interestingly, the awareness for this problem seems to be more developed in the group of family doctors than in the surgical community, since the majority of non-indicated SUPs not discontinued on discharge were stopped during the 3 months following the end of the hospital stay. This is different from older reports, where ASM started without adequate indication during hospitalisation was still present in 46–79.4% of patients 3 months after discharge [15, 20].

There is an important fraction of patients admitted to the surgical department already receiving AST prescribed previously by their general practitioner. In our patient cohort, 39.1% of patients had AST in their home medication. This prevalence is slightly superior to those reported in internal medicine patients (10.7–33.1%) [14–16, 18] [12, 28]. Although no indication for this treatment could be determined upon review of the patient charts, AST was continued in virtually all patients during the hospitalisation, and prescription was continued in the discharge letter in the majority of patients (93%). This indicates that critical appraisal of the indication for this class of medication is not sufficiently developed in order to reduce the wide spread over-prescription of AST. In this setting, pharmacist-driven protocols or participation of pharmacists on ward rounds have been shown to effectively reduce the overuse of AST during and after hospitalisation [41].

In summary, despite the vigorous debate on AST over-prescription for SUP led in the current literature, SUP is still widely practised in surgical patients in the non-ICU setting. The official guideline on SUP was published more than 20 years ago, only briefly addresses SUP in the non-ICU setting and advises against routine SUP in adult medical and surgical patients in non-ICU settings [26]. However, various constellations involving risk medications, polypharmacy and multimorbidity require more practicable recommendations on this issue. The development and implementation of local standard operating procedures addressing the issue of SUP as well as pharmacist-driven protocols may be successful tools to reduce the inappropriate overmedication with AST.

## Supplementary Information

Below is the link to the electronic supplementary material.

Supplementary file1 (DOCX 20 KB)
