# Supplementary material for: Current practice of stress ulcer prophylaxis in a surgical patient cohort in a German university hospital
Source: Langenbecks Arch Surg. 2021 Sep 14;406(8):2849–59. doi: 10.1007/s00423-021-02325-3 (PMC8803691; doi:10.1007/s00423-021-02325-3)
Supplement: Supplementary file 1 — Supplementary file1 (DOCX 20 KB) [file 423_2021_2325_MOESM1_ESM.docx]

|  |  | Patients admitted to the hospital w/o ASM **with SUP** during hospitalisation | Patients admitted to the hospital w/o ASM **w/o SUP** during hospitalisation | p |
| --- | --- | --- | --- | --- |
| Thoracic pathologies | | n=59 | n=23 |  |
| Thyroid benign  Thyroid malign  Lung benign  Lung malign  Thorax others | | 37(78,7)  5(83,3)  5(83,3)  7(46,7)  5(62,5) | 10(21,3)  1(16,7)  1(16,7)  8(53,3)  3(37,5) | 0,139 |
| Vascular pathologies | | n=39 | n=54 |  |
| Vascular arterial  Vascular venous  Vascular divers | | 22(44,9)  2(13,3)  15(51,7) | 27(55,1)  13(86,7)  14(48,3) | 0,042 |
| Visceral pathologies/pathologies of the abdominal wall | | n=149 | n=295 |  |
| Stomach benign  Stomach malign  Colorectal benign  Colorectal malign  Small intestine  Hepatobiliary benign  Hepatobiliary malign  Hernia  Appendizitis  Cholecystitis/cholecystolithiasis  Visceral others benign  Visceral others malign | | 3(100)  3(100)  24(37,5)  25(53,2)  6(85,7)  1(50)  7(100)  9(12,5)  17(37)  21(29,6)  32(29,1)  1(8,3) | 0  0  40(62,5)  22(46,8)  1(14,3)  1(50)  0  63(87,5)  29(63)  50(70,4)  78(70,9)  11(91,7) | 0,0 |

***Supplementary Table 1:*** Detailed diagnosis at discharge of patients admitted to the hospital without previous acid suppressive medication. Patients being started on SUP during hospitalisation are compared to those without SUP. Percentages are given within each diagnostic entity.
